# Apical inflow is associated with increased energy loss during left ventricular diastole in patients with a repaired atrioventricular septal defect: a 4D flow MRI study

**DOI:** 10.1186/1532-429X-18-S1-P24

**Published:** 2016-01-27

**Authors:** Mohammed SM ElBaz, Arno Roest, Emmeline Calkoen, Patrick J de Koning, Boudewijn PF Lelieveldt, Rob J van der Geest, Jos J Westenberg

**Affiliations:** 1 Division of Image Processing, Radiology, Leiden University Medical Center (LUMC), Leiden, Netherlands; 2 Department of Paediatric Cardiology, Leiden University Medical Center, Leiden, Netherlands; 3 Department of Intelligent Systems, Delft University of Technology, Delft, Netherlands

## Background

Patients after atrioventricular septal defect (AVSD) repair have aberrant atrioventricular valve morphology resulting in a disorganized left ventricular (LV) inflow [1] with an increased amount of inflow reaching the apical level of the LV and retaining there during the subsequent systole [1].This may elevate energy loss in LV flow due to friction. We aimed to evaluate the association between the percentages (retained) apical inflow with viscous energy loss during diastole in AVSD-repaired patients compared to healthy controls using 4D flow MRI.

## Methods

21 AVSD-repaired patients (age: 33 ± 9 years) and 16 healthy controls (age: 35 ± 11 years, p = 0.52) underwent free-breathing whole-heart 4D flow MRI at 3T(VENC =150 cm/s in all directions, spatial resolution 2.3 × 2.3 × 3.0-4.2 mm^3^, 30 retrospectively reconstructed phases over a cardiac cycle). The workflow in [1] was used to compute, relative to total inflow, percentages of apical inflow (AI%: inflow that reaches apical level during diastole), and retained apical inflow (RAI%: part of apical inflow that is not ejected during subsequent systole). The LV was segmented and the total non-turbulent viscous energy loss was computed over diastole (EL_diastole in Joule (J)) using the Navier-Stokes energy equations [2]. The 95%CI (confidence interval) for AI% and RAI% was computed from healthy controls. Patients were then classified as: below or above upper limit of 95%CI of AI%, and below or above upperlimit of 95%CI of RAI%. Parameters were compared using Student's t-test. Association between EL_diastole with AI% and RAI% was evaluated using Pearson's correlation.

## Results

**Figure 1:**
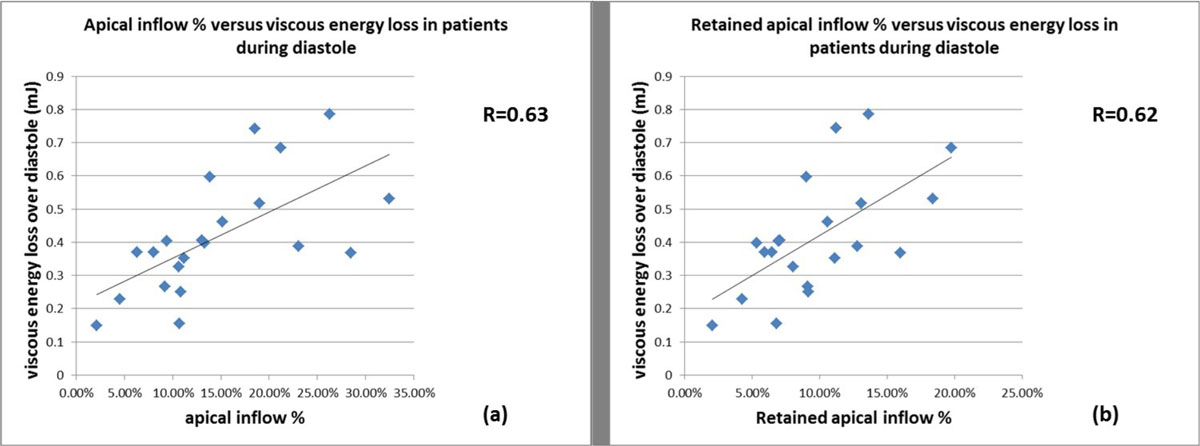
**Correlation between viscous energy loss over diastole (EL_diastole in milliJoules (mJ)) with (a) the percentage of apical inflow (AI%: i.e. percentage of inflow that reaches to the apex of the LV during diastole) and (b) the percentage of retained apical inflow (RAI%: i.e. fraction of the apical inflow that is not ejected during subsequent systole) in the AVSD-repaired patients. The solid line corresponds to the regression line**.

The 95%CI of healthy controls was [6.23%, 11.66%] for AI% and [3.9%, 7.11%] for RAI%. In 11 patients AI% was above 95%CI (20.4 ± 6.59%). In these patients, EL_diastole was significantly increased relative to controls (patients: 0.53 ± 0.15 mJ vs controls: 0.31 ± 0.13 mJ, p < 0.001) while the 10 patients below upperlimit 95%CI showed no significant difference relative to controls (patients: 0.44 ± 0.17 mJ, p = 0.07). In 13 patients RAI% was above 95%CI (18.46% ± 7.5%). These patients presented a significant increase in EL_diastole relative to controls (0.48 ± 0.18 mJ, p = 0.005) and to the 8 patients below upperlimit 95%CI (0.31 ± 0.11, p = 0.02). These 8 patients showed no significant difference to controls' EL_diastole (p = 0.94). In patients, EL_diastole was significantly positive correlated with AI% (R = 0.63, p = 0.002) and with RAI% (R = 0.62, p = 0.002) (Figure 1).

## Conclusions

This is the first study to reveal the adverse impact of increased (retained) apical inflow on LV energy loss. Elevated viscous energy loss in patients reflects increased blood flow friction. This could potentially result in apical blood stagnation. Future-studies are needed to assess the impact of the reported association on LV function. The provided 4D flow workflow may permit a non-invasive tool to assess blood inflow efficiency in different patient groups.
